# Biopsychosocial medicine research trends: connecting clinical medicine, psychology, and public health

**DOI:** 10.1186/s13030-020-00204-9

**Published:** 2020-12-08

**Authors:** Mutsuhiro Nakao, Gen Komaki, Kazuhiro Yoshiuchi, Hans-Christian Deter, Shin Fukudo

**Affiliations:** 1 Department of Psychosomatic Medicine, School of Medicine, International University of Health and Welfare, 4-3, Kozunomori, Narita-shi, Chiba, 286-8686 Japan; 2 Fukuoka International University of Health and Welfare, Faculty of Medical Science, Fukuoka, Japan; 3 Department of Psychosomatic Medicine, School of Medicine, The University of Tokyo, Tokyo, Japan; 4 Medical Clinic, Psychosomatics, Charité Universitätsmedizin Berlin, Campus Benjamin Franklin, Hindenburgdamm, Berlin, Germany; 5 Department of Behavioral Medicine, Tohoku University Graduate School of Medicine & Department of Psychosomatic Medicine, Tohoku University Hospital, Sendai, Japan

## Growth of biopsychosocial medicine

The journal *BioPsychoSocial Medicine* was launched in January 2007, nearly 13 years ago. This is a peer-reviewed online journal that encompasses all aspects of the interrelationships between the biological, psychological, social, and behavioral elements of health and disease [1]. The journal emphasizes a biopsychosocial approach to illness and health, covering the behavioral sciences, social sciences, neuroscience, stress physiology and epidemiology, psychoneuroendocrinology/immunology, gut-brain axis, psycho-cardiology and psycho-oncology, all of which are associated with mind–body interactions and psycho social interventions including psychosomatic/behavioral therapeutic approach..

As of October 2020, 331 articles have been published, comprising 222 original research reports (67.1%), 63 reviews (19.0%), 25 case reports (7.6%), 14 editorials (4.2%), and seven other articles (2.1%). When all published articles were assigned to three main areas of study, biological/psychosomatic medicine, psychology, and public health, 142 (42.9%) articles were categorized in the biological/psychosomatic domain, 120 (36.3%) in the psychological area, 51 (15.4%) in public health, and 18 (5.4%) as “others”. The main topics in each area are shown in Table 1. A variety of psychosomatic illnesses have been investigated, including eating disorders, irritable bowel syndrome, chronic pain, chronic fatigue syndrome, coronary heart diseases, and allergic diseases. Articles related to women’s and children’s health and series in several clinical fields like pediatrics, gynecology, and dentistry were initiated. Cognitive behavioral therapies, relaxation training, and special treatments as Yoga or Kampo medicine have often been employed with such psychosomatic illnesses, and were carefully evaluated. Social factors such as work stress and over-adaptation have been shown to be associated with mind/body health.

**Table 1:** Examples of research topics published in *BioPsychoSocial Medicine* from January 2007 to October 2020

Research area	Research topics
Biological	Physiological mechanisms of psychosomatic illnesses: chronic fatigue syndrome, dizziness and tinnitus, eating disorders, irritable bowel syndrome, migraine, oral health, premenstrual symptoms, tension-type headache Psychosomatic treatments: Kampo medicine, psychosomatic basic care, Yoga, Physiological markers: auditory evoked potential, ecological momentary assessment, heart rate variability, neuroimaging, salivary amylase, very long chain fatty acids
Psychological	Psychopathological concepts: alexithymia, alexisomia, somatosensory amplification, somatosensory catastrophic thought Psychological treatments: behavioral activation therapy, cognitive behavioral therapy, mindfulness
Social	Social science and medicine: adaptation, child abuse, education and training in behavioral/psychosomatic medicine, social support, health literacy and health communication, suicide problem, Occupational health: effort–reward imbalance at work, work engagement, work stress

## Recent interest in biopsychosocial medicine

Interest in biopsychosocial medicine has grown recently. For example, according to tabulations by Springer Nature, articles in this journal were accessed 197,797 times during 2019. This translates to an average of 16,483 accesses per month, with frequencies ranging from 12,345 in January to 21,324 in December 2019. Major visits by geography were as follows; United States (35%), United Kingdom (10%), Japan (9%), India (7%), Australia (6%), Canada (5%), Philippines (3%), Germany (1%), Netherlands (1%), Italy (1%), Ireland (1%), and Indonesia (1%).

The top 10 articles, selected from among those published during 2007–2019 and most frequently accessed by Internet in 2019, are shown in Table 2. It is surprising that the first two original articles, one by Decety et al. (2007) and one by Sugimoto et al. (2009), are still accessed often, although it is now more than 10 years since their publication. When limiting the analyses to the articles published in the past 3 years, i.e., 2017–2019, the most frequently accessed article, authored by Hirayama et al. (2019), was a case series reporting the treatment effects of behavioral activation therapy for depression and anxiety in cancer patients (Table 3). No article published in 2017 and only one published in 2018 were identified among the top 10 in the 2019 access ranking.

**Table 2:** Top 10 articles published in *BioPsychoSocial Medicine* in the period from 2007 to 2019 based on the numbers of Internet access during 2019

Authors	Title	Article type	Publication year	Access number in 2019
Jean Decety et al.	The empathic brain and its dysfunction in psychiatric populations: implications for intervention across different clinical conditions	Review	2007	9857
Tomoko Matsushita et al.	A large-scale survey of adverse events experienced in yoga classes	Original	2015	6398
Takatoshi Hirayama et al.	Behavioral activation therapy for depression and anxiety in cancer patients: a case series study	Case report	2019	5447
Takakazu Oka et al.	Psychological stress contributed to the development of low-grade fever in a patient with chronic fatigue syndrome: a case report	Case report	2013	4712
Mizuho Hosogi et al.	Importance and usefulness of evaluating self-esteem in children	Review	2012	4265
Nagisa Sugaya et al.	Bio-psychosocial factors of children and adolescents with Internet gaming disorder: a systematic review	Review	2019	3906
Trang T. H. Tu et al.	Current management strategies for the pain of elderly patients with burning mouth syndrome: a critical review	Review	2019	3189
Mutsuhiro Nakao	Board games as a promising tool for health promotion: a review of recent literature	Review	2019	3101
Hiroshi Sugimoto et al.	Iron deficiency anemia induced by magnesium overuse: a case report	Case report	2019	2870
Koreaki Sugimoto et al.	The effectiveness of the Uchida–Kraepelin test for psychological stress: an analysis of plasma and salivary stress substances	Original	2009	2756

**Table 3:** Top 10 articles published in *BioPsychoSocial Medicine* in the past 3 years (2017 to 2019) that were most frequently accessed through the Internet during 2019

Authors	Title	Article type	Publication year	Access number in 2019
Takatoshi Hirayama et al.	Behavioral activation therapy for depression and anxiety in cancer patients: a case series study	Case report	2019	5447
Nagisa Sugaya et al.	Bio-psychosocial factors of children and adolescents with Internet gaming disorder: a systematic review	Review	2019	3906
Trang T. H. Tu et al.	Current management strategies for the pain of elderly patients with burning mouth syndrome: a critical review	Review	2019	3189
Mutsuhiro Nakao	Board games as a promising tool for health promotion: a review of recent literature	Review	2019	3101
Hiroshi Sugimoto et al.	Iron deficiency anemia induced by magnesium overuse: a case report	Case report	2019	2870
Rasool Kawyannejad et al.	General health of students of medical sciences and its relation to sleep quality, cell phone overuse, social networks and Internet addiction	Original	2019	2533
Filiberto Toledano-Toledano et al.	Psychosocial factors related with caregiver burden among families of children with chronic conditions	Original	2019	2359
Toru Takahashi et al.	Changes in depression and anxiety through mindfulness group therapy in Japan: the role of mindfulness and self-compassion as possible mediators	Original	2019	2236
Takakazu Oka et al.	Changes in fatigue, autonomic functions, and blood biomarkers due to sitting isometric yoga in patients with chronic fatigue syndrome	Original	2018	1783
Yohei Okawa et al.	Specific foods can reduce symptoms of irritable bowel syndrome and functional constipation: a review	Review	2019	1597

Concerning the number of journal citations, Web of Science, the top 20 articles were shown in Table 4 [2]. Half of them were review articles, and the remaining half were original research ones. The most recent articles listed in the table were one by Kano et al. (2013) and one by Moriguchi et al. (2013), both of which were reviews addressing brain function of alexithymia.

**Table 4:** Top 20 articles published in *BioPsychoSocial Medicine,* based on the number of journal citations, Web of Science, in October, 2020 [2]

Authors	Title	Article type	Publication year	Citation
Jean Decety, et al.	The empathic brain and its dysfunction in psychiatric populations: implications for intervention across different clinical conditions	Review	2007	210
Louis T van Zyl, et al.	Effects of antidepressant treatment on heart rate variability in major depression: A quantitative review	Review	2008	73
Alexander Hansel, et al.	The ventro-medial prefrontal cortex: a major link between the autonomic nervous system, regulation of emotion, and stress reactivity?	Review	2008	72
Michiko Kano, et al.	The alexithymic brain: the neural pathways linking alexithymia to physical disorders	Review	2013	71
Michael P Muehlenbein, et al.	The costs of dominance: testosterone, cortisol and intestinal parasites in wild male chimpanzees	Original	2010	71
Yoshiya Moriguchi, et al.	Neuroimaging studies of alexithymia: physical, affective, and social perspectives	Review	2013	62
Daisuke Nishi, et al.	Posttraumatic growth, posttraumatic stress disorder and resilience of motor vehicle accident survivors	Original	2010	62
Mutsuhiro Nakao, et al.	Clinical application of somatosensory amplification in psychosomatic medicine	Review	2007	60
Yoshiya Moriguchi, et al.	Age and gender effect on alexithymia in large, Japanese community and clinical samples: a cross-validation study of the Toronto Alexithymia Scale (TAS-20)	Original	2007	59
Sidney Bloch, et al.	Psychological adjustment of men with prostate cancer: a review of the literature	Review	2007	56
Nahathai Wongpakaran, et al.	The Thai version of the PSS-10: An Investigation of its psychometric properties	Original	2010	56
Mutsuhiro Nakao	Work-related stress and psychosomatic medicine	Review	2010	48
Hirono Ishikawa, et al.	Health literacy and health communication	Review	2010	44
Masayo Kojima	Alexithymia as a prognostic risk factor for health problems: a brief review of epidemiological studies	Review	2012	44
Antonina A Mikocka-Walus, et al.	Does psychological status influence clinical outcomes in patients with inflammatory bowel disease (IBD) and other chronic gastroenterological diseases: An observational cohort prospective study	Original	2008	41
Bo Simonsson, et al.	Psychosomatic complaints and sense of coherence among adolescents in a county in Sweden: a cross-sectional school survey	Original	2008	41
Motoyori Kanazawa, et al.	Translation and validation of a Japanese version of the irritable bowel syndrome-quality of life measure (IBS-QOL-J)	Original	2007	39
Mariko Ogawa, et al.	Evaluation of factors associated with the anxiety and depression of female infertility patients	Original	2011	39
Kazufumi Yoshihara, et al.	Profile of mood states and stress-related biochemical indices in long-term yoga practitioners	Original	2011	38
Takakazu Oka, et al.	Rikkunshi-to attenuates adverse gastrointestinal symptoms induced by fluvoxamine	Original	2007	35

## Future directions of biopsychosocial medicine

Editors and colleagues have regularly carried out thematic series addressing psychosomatic medicine to facilitate researchers’ submission of reports focusing on a variety of biopsychosocial topics (Table 5). Interestingly, behavioral medicine was featured twice in the journal in 2016. Ours is an interdisciplinary field combining medicine, psychology, and social science, and the practice of psychosomatic medicine is closely related to that of behavioral medicine, particularly in terms of the biopsychosocial aspects of health.

**Table 5:** Examples of thematic series published in *BioPsychoSocial Medicine*

Proposed theme	Editor in charge	Publication year
Perspectives of public health in bio-psycho-social medicine	Mutsuhiro Nakao	2010
Alexisomia: a shift in focus from alexithymia	Gen Komaki	2012
Bio-psycho-social medicine in pediatrics	Hidetaka Tanaka	2012
Focusing psychosocial interventions in chronic somatic disease—new tasks and strategies for conducting psychosomatic treatment studies	Hans-Christian Deter	2012
Integrating kampo into psychosomatic medical practice	Takakazu Oka	2014
The gut–brain axis: emerging evidence in health and disease	Cross-journal collection	2014
Allergic disease and psychosocial stress	Nobuyuki Sudo	2015
Current status of eating disorders: general and special population studies	Cross-journal collection	2015
History, concepts and aims of international societies in psychosomatic and behavioral medicine	Hans-Christian Deter	2016
Psychosomatic dentistry	Akira Toyofuku	2016
Recent advances in psychosomatic obstetrics and gynecology in Japan	Masakazu Terauchi	2016
The meaning of behavioral medicine in the psychosomatic field	Mutsuhiro Nakao	2016
Somatic manifestation of distress: clinical medicine, psychological, and public health perspectives	Mutsuhiro Nakao	2017
Psycho-oncology in the Asia–Pacific area	Kazuhiro Yoshiuchi	2017
Effects of board games on health education and promotion	Mutsuhiro Nakao	2019

More than 40 years have passed since Engel developed a biopsychosocial model that went beyond traditional biochemical models of clinical medicine [3], and more than 1500 articles have been published, according to the PubMed search using a text word of “biopsychosocial-model”. The Japanese Society of Psychosomatic Medicine defines psychosomatic illness as any physical condition with organic or functional damage affected by psychosocial factors in its onset or development [4], and the biopsychosocial model is useful in improving clinical outcomes of such psychosomatic illnesses and a variety of chronic diseases, through creating awareness on the interactions among biological, psychological, sociocultural, and spiritual factors, and to enhance self-management of illness conditions through multidisciplinary approach of patient care and other medical settings [5]. Although we know both favorable and critical opinions in the pre-existing literature, we still believe that the biopsychosocial model continues to offer valuable insights into clinical practice, medical education, and psychosomatic research and that it should be further developed to treat and prevent stress-related conditions.

According to the recent report [6], mind-body approach, including Yoga, meditation, or other Eastern medicine techniques, can be a helpful adjunct in managing stress-related noncommunicable diseases by fostering resilience through self-care. BioPsychoSocial Medicine is the unique journal locating in Asia but disseminating the importance of psychosomatic medicine all over the world. Such successive activities will help mutual understanding and fusion of East and West in terms of mind-body connections of health. The editors welcome high-quality research clarifying mind/body relationship as they affect and are affected by health behaviors and social life in humans.

## Data Availability

Obtained data and materials were based on information about the journal and are available only to editorial board members.
